# Correction of a Clipping

**Published:** 1892-08

**Authors:** Williamson Bryden


*To the Editors of the Journal of Comparative Medicine and Veterinary Archives.*

GENTLEMEN :—I have just read in the *American Veterinary Review* a clipping from the *New York Medical Journal*, which is so wide of the mark that it demands correction. It is headed "A worthy sanitary feat quietly accomplished," and reads as follows : "It is alleged for General Rusk that he has greatly improved the treatment of cattle exported to Europe for food purposes. The mortality among them at sea resulting from cruelty, want of water, etc., was formerly stated at sixteen per cent., while at the present time, it is one per cent., etc., etc."

Such a misstatement is evidently made to flatter General Rusk, for he certainly deserves well from the veterinary profession, which he has patronized in the most liberal manner; but that he will care for such a compliment one can hardly conceive of a gentleman in his high position.

The statement is probably made by some enthusiastic political admirer of his, who knows very little about the subject, for it is misleading and untrue, as the records of the regular lines and the insurance rates prove. During the last two years, when General Rusk has had charge of the Agricultural Department at Washington, rates of insurance have not improved more than a half of one per cent., and this has been due to the splendid achievements of the ship owners and builders, and the judgment and care of the masters and officers of the ships.

If praise is due to any one at Washington, it is to Dr. Salmon, for his share in successfully controlling Texas or southern fever, which was accomplished before the advent of General Rusk at the Bureau of Animal Industry. In the summer months, when this disease prevailed, underwriters demanded from five to seven per cent. Indeed, they did not care for the risks at any rate. For the last five or six years risks have never been more than from one and a half to two per cent., which proves the absurdity of figures like sixteen per cent. For years, on some of the regular steamship lines, their losses have frequently run as low as one-quarter, one-third and one-half per cent. per annum.

Had our railroads been treated in the arbitrary manner in which the steamships have been dealt with by this government, and forced to make equal improvements, it would have been much more creditable to General Rusk's reputation for fairness ; the losses at present being almost invariably the result of injuries received on the cars, from their slipping down on wet, *uncleated* carfloors, when the air-brakes are suddenly applied, the animals are then thrown on top of each other into one end of the car, this is especially the case with large, heavy distillers, a class of animals that are ill adapted for shipment in the cars in hot weather, unless handled and attended with the greatest care ; it is no unusual thing to see half the car load down and bruised and lame ; division boards and cleats are quite as essential on cars as on board ship, and that the railroads and stock yards are allowed to do "the go-as-you-please" act, while foreign ships are forced to submit to restrictions that would not be thought of if they belonged to the United States, is neither creditable nor fair.

WILLIAMSON BRYDEN.

